# Angiogenesis-Inflammation Cross Talk in Diabetic Retinopathy: Novel Insights From the Chick Embryo Chorioallantoic Membrane/Human Vitreous Platform

**DOI:** 10.3389/fimmu.2020.581288

**Published:** 2020-09-29

**Authors:** Sara Rezzola, Alessandra Loda, Michela Corsini, Francesco Semeraro, Tiziana Annese, Marco Presta, Domenico Ribatti

**Affiliations:** ^1^Department of Molecular and Translational Medicine, School of Medicine, University of Brescia, Brescia, Italy; ^2^Eye Clinic, Department of Neurological and Vision Sciences, University of Brescia, Brescia, Italy; ^3^Department of Basic Medical Sciences, Neurosciences, and Sensory Organs, University of Bari Medical School, Bari, Italy; ^4^Italian Consortium for Biotechnology (CIB), Unit of Brescia, Brescia, Italy

**Keywords:** angiogenesis, inflammation, vitreous, chick embryo CAM, diabetic retinopathy

## Abstract

Pathological angiogenesis of the retina is a key component of irreversible causes of blindness, as observed in proliferative diabetic retinopathy (PDR). The pathogenesis of PDR is complex and involves vascular, inflammatory, and neuronal mechanisms. Several structural and molecular alterations associated to PDR are related to the presence of inflammation that appears to play a non-redundant role in the neovascular response that characterizes the retina of PDR patients. Vascular endothelial growth factor (VEGF) blockers have evolved over time for the treatment of retinal neovascularization. However, several limitations to anti-VEGF interventions exist. Indeed, the production of other angiogenic factors and pro-inflammatory mediators may nullify and/or cause resistance to anti-VEGF therapies. Thus, appropriate experimental models are crucial for dissecting the mechanisms leading to retinal neovascularization and for the discovery of more efficacious anti-angiogenic/anti-inflammatory therapies for PDR patients. This review focuses on the tight cross talk between angiogenesis and inflammation during PDR and describe how the chick embryo chorioallantoic membrane (CAM) assay may represent a cost-effective and rapid *in vivo* tool for the study of the relationship between neovascular and inflammatory responses elicited by the vitreous humor of PDR patients and for the screening of novel therapeutic agents.

## Introduction

Retinal and choroidal neovascularization are the leading causes of visual impairment in various ocular pathologies, including retinal vein occlusion, age-related macular-degeneration, retinopathy of prematurity and diabetic retinopathy (DR).

DR is one of the main complications of diabetes mellitus and it represents the major cause of vision loss in the working-age population (1). At present, 463 million adults are estimated to be living with diabetes worldwide, a number projected to rise to 700 million by 2045 (2). Currently, DR affects more than 93 million people in the world with an overall prevalence close to 35% of the diabetic population (3). In the earlier stages, the disease manifests as non-proliferative microaneurysms; then, it progresses to proliferative diabetic retinopathy (PDR). Hallmarks of PDR are the presence of hard and soft exudates, neovascularization and hemorrhages. The retinal microvasculature is progressively damaged by the disease, resulting in various events such as retinal ischemia, upregulation of hypoxia inducible factor-1 (HIF-1), and vascular endothelial growth factor (VEGF) secretion, possibly progressing to PDR, which is diagnosed according to the presence of vascular lesions (e.g., preretinal or vitreous hemorrhages or neovascularization) (4).

Inflammation and angiogenesis are two of the main factors that contribute to PDR. During the disease, inflammation and neovascularization establish a strict cross talk, with inflammation promoting neovascularization and *vice versa* [see (5–8) and references therein]. Interestingly, clinical evidence shows a lower occurrence of DR in diabetic patients treated with salicylates for rheumatoid arthritis (9). Accordingly, anti-inflammatory drugs could be beneficial for managing retinal neovascularization. Indeed, the progression of pathological neovascularization and of diabetic macular edema may be reduced by the administration of corticosteroids (e.g., triamcinolone acetonide) via intravitreal injection. Even though, corticosteroids could be effective in improving or at least stabilizing visual acuity, these results are often temporary and administration of corticosteroids may be associated with adverse effects, such as increased intraocular pressure and cataract formation (10–12).

Laser photocoagulation is a widely used technique for treating retinal neovascularization, allowing long-term regression. However, the identification of VEGF as a key mediator in the pathogenesis of DR, able to promote both angiogenesis and vascular permeability, led to the establishment of anti-VEGF agents as an alternative line of treatment (4). Clinical and experimental evidence suggests that intraocular levels of VEGF are increased during retinal ischemia, resulting in the breakdown of the blood-retina barrier, enhanced vascular permeability, and neovascularization (13).

A recent meta-analysis of aggregate data has indicated that anti-VEGF pharmacotherapy is associated with superior visual acuity outcomes and less PDR-related complications when compared to retinal laser photocoagulation (14). However, limitations do exist in the use of anti-VEGF agents. Indeed, due to their brief duration of action, anti-VEGF drugs need to be frequently administered *via* intravitreal injection, possibly resulting in adverse side effects (i.e., endophthalmitis and ocular inflammation). Furthermore, a large percentage of patients do not respond to anti-VEGF drugs or exhibit a poor response. Supposedly, this limited efficacy may depend on the activation of other pathways promoting ocular angiogenesis as a consequence of the local production of various pro-angiogenic and pro-inflammatory factors [reviewed in (15–17)].

Therefore, a better knowledge of the pathogenesis of DR is required, in order to clarify the relationship between inflammation and angiogenesis during the disease progression. Indeed, a better understanding of their role in the disease could allow for the identification of novel anti-inflammatory approaches targeting retinal angiogenesis. In this frame, the implementation of new methods that could allow the discovery of novel strategies targeting molecular pathways involved in ocular neovascularization is essential. To achieve this aim, many pharmacological studies have been carried out in various *in vitro* and *ex vivo* assays, suitable for the screening of small anti-angiogenic compounds (16, 18). In addition, mouse models have been established in order to investigate retinal angiogenesis (19, 20). However, the use of these models is hindered by various limitations (21).

The chick embryo chorioallantoic membrane (CAM) has been proposed as a valid alternative animal model for the investigation of the mechanisms underlying physiological and pathological angiogenesis (22). This review highlights the use of the CAM as a model system for the study of the cross talk between angiogenesis and inflammation in PDR and for the screening of anti-angiogenic/anti-inflammatory molecules to be employed for the treatment of angiogenesis-dependent eye diseases.

## Angiogenesis and Inflammation in Diabetic Retinopathy

Angiogenesis is a complex multi-step process. Various events are necessary for angiogenesis to occur, including the interaction between cell surface receptors, soluble factors, and extracellular matrix components. Several cell types are also required, with endothelial cells playing a major role (23).

The formation of neovessels has been thoroughly investigated and described in several insightful reviews (24–28). Briefly, hypoxia promotes the release of angiogenic factors, such as VEGF, responsible for inducing the detachment of pericytes from the vessel wall, which weakens the interactions among endothelial cells and increases vascular permeability (23). Moreover, pro-angiogenic molecules directly increase vascular permeability by disrupting adherens junctions and by inducing the phosphorylation of vascular endothelial-cadherin, thus allowing serum proteins extravasation from the vascular lumen (29). Pro-angiogenic mediators stimulate the activation of quiescent endothelial cells, which alter their morphology and acquire a “pro-angiogenic phenotype.” Once activated, endothelial cells proliferate and migrate into the stroma, following a chemotactic gradient provided by the angiogenic stimulus (30). Finally, the neovessels complete their maturation process by the deposition of a basal membrane and the recruitment of pericytes/smooth muscle cells. After all these steps have been accomplished, the production of pro-angiogenic mediators decreases, the neovessels are remodeled by the blood flow itself, and endothelial cells return to their quiescent condition (31).

During diabetes, hyperglycemia acts on retinal endotelium, promoting the activation of interconnected biochemical pathways, including the polyol (sorbitol-aldose reductase) (32) and hexosamine (33) pathways, enhanced production of advanced glycation end products (34) and reactive oxygen species (ROS) (35), and activation of protein kinase C (36, 37), poly(ADP-ribose) polymerase (38), and of the renin-angiotensin system (39). All of these events contribute to increasing oxidative stress, which, in turn, triggers neovascularization, inflammation, and early neurodegeneration. Moreover, hyperglycemia affects retinal mitochondria, which become dysfunctional. Consequently, the production of ROS is increased, damaging DNA, promoting the release of cytochrome C, and resulting in endothelial cell apoptosis (40). Another important feature of the vascular dysfunction that occurs during DR is the loss of retinal pericytes, which further destabilizes endothelial cells and alters perfusion (41). The tight interaction between pericytes and endothelium is disrupted by the progressive thickening of the basement membrane that, together with systemic and local hypertension, promotes pericyte apoptosis.

These hyperglycemia-induced alterations are considered one of the primary events in the pathogenesis of DR and they are followed by other dysfunctions, such as retinal hyperpermeability, thickening of the basal endothelial membrane, and activation of a strong inflammatory response.

Another hallmark of DR is the presence of micro-occlusions in the retinal microvasculature (42). Endothelial cells upregulate the expression of the intracellular adhesion molecule 1 (ICAM-1), which is responsible for mediating the adhesion of leukocytes to the endothelium (43). The constriction of major arteries and arterioles leads to areas of decreased perfusion associated with an upregulation of HIF-1, which levels are elevated in the vitreous of PDR patients (44, 45). HIF-1 upregulates several growth factors, cytokines, and chemokines, leading to retinal neovascularization (46). These HIF-1-regulated factors include various pro-angiogenic molecules, such as VEGF, erythropoietin, fibroblast growth factor 2 (FGF2), insulin-like growth factor-1, stromal cell-derived factor-1, platelet-derived growth factor, tumor necrosis factor α (TNFα) and interleukins (ILs) (17, 47–49). In addition, many anti-angiogenic mediators are downregulated, including angiostatin and pigment epithelium-derived factor and decreased levels of these molecules have been reported in the vitreous of diabetic patients (50).

A tight cross talk between inflammation and angiogenesis takes place in several physiological and pathological conditions (51, 52). Inflammatory cells are responsible for the production of various molecules, including growth factors, cytokines, and proteases. All of these mediators contribute to neovessel formation (53). Moreover, activated endothelial cells express pro-inflammatory molecules that mediate the recruitment and the activation of white blood cells (54, 55). Several signaling pathways are shared by neovascularization and inflammation processes (56). Indeed, various chemokines might exert a double function by promoting leukocyte adhesion to the endothelium and stimulating endothelial cell proliferation (57). In addition, several pro-inflammatory cytokines, including IL6, IL1α, IL1β, osteopontin, high mobility group box-1, and TNFα, may directly activate angiogenesis by acting on endothelial cells. These same cytokines also promote angiogenesis indirectly by activating the production of more pro-angiogenic factors by leukocytes and endothelium (58–60). Conversely, endothelial cells stimulated by the pro-angiogenic factors VEGF and angiopoietin-1 increase the expression of cell adhesion molecules, as well as the production of inflammatory factors (61, 62). A further example of the cross talk that occurs between angiogenesis and inflammation is provided by the capacity of pro-inflammatory stimuli to induce the upregulation of *HIF-1*α gene expression *via* the activation of the canonical nuclear factor κB (NF-κB) pathway, a key regulator of innate immune, inflammatory and angiogenic responses (63). In addition, oxygen-sensing hydroxylases may confer hypoxic sensitivity to both HIF and NF-κB pathways concurrently (64). Thus, a tight interaction exists between HIF and NF-κB signaling that leads to the production of inflammatory and angiogenic mediators under hypoxic conditions, including VEGF (65).

Inflammation is a crucial event for the development of DR. It is especially relevant during the initial stages of the disease, when inflammation activates transcriptional factors and induces the increased expression of both pro-inflammatory and pro-angiogenic mediators (66, 67). Retinal inflammation is closely associated with neovascularization. Indeed, during inflammation, retinal microglia become activated and release cytokines and pro-angiogenic mediators (68) responsible for the maintenance of chronic inflammation in the retina (7, 69). Prolonged inflammation is extremely detrimental and it contributes to damaging retinal vasculature, promoting the formation of neovessels as well as the development of macular edema (7, 70). Moreover, inflammation may be involved in retinal neurodegeneration, which is frequently observed in DR patients (7, 71). New insights into the exact role of inflammation in the pathogenesis of DR may allow for the identification of new molecular pathways and for the discovery of novel therapeutic targets. The association of anti-angiogenic and anti-inflammatory drugs may therefore be beneficial for treating DR (71–73).

## The Chick Embryo Chorioallantoic Membrane

**Figure 1:**
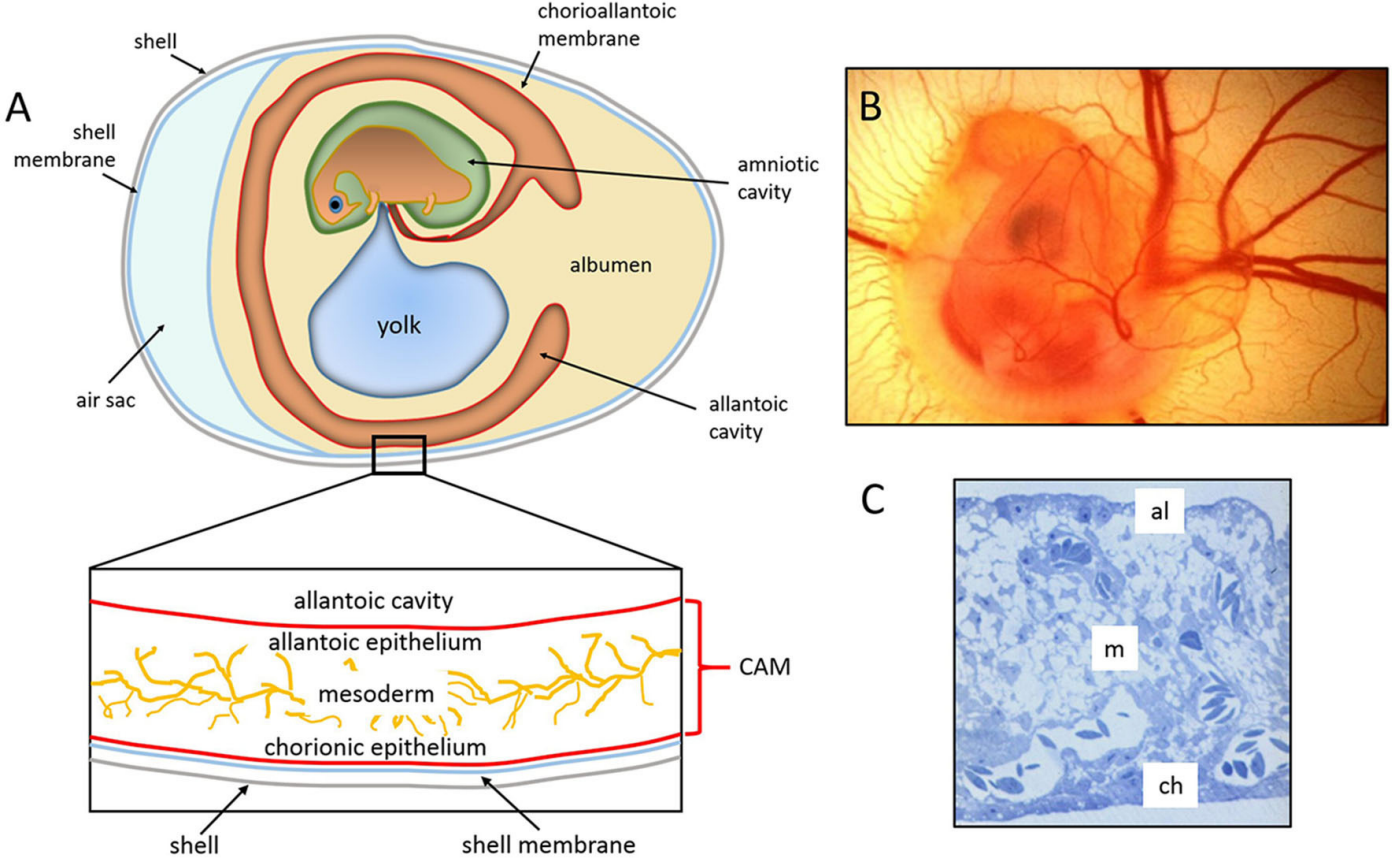
The chick embryo and its chorioallantoic membrane. **(A)** Schematic drawing of the general structure of a 5 day old chick embryo in the egg and the three-tissue layers of the chick chorioallantoic membrane (CAM). **(B)** Semithin section of the CAM of a 12 day old chick embryo showing the chorionic epithelium (ch), the vascularized mesoderm (m), and the allantoic epithelium (al). **(C)** 5 day old chick embryo photographed *in ovo* [**(B,C)**, reproduced from (75)].

The chick embryo CAM is a vascular membrane formed by the fusion of the mesodermal layers, the allantois, and the chorion that appears at day 3–4 of incubation. It consists of three layers, ectoderm (originating from the chorion and attached to the shell membrane), mesoderm (represented by the fusion of the somatic mesoderm from the chorion and the splanchnic mesoderm from the allantois), and endoderm (originating from the allantois and facing up the allantoic cavity) (74). The middle mesodermal layer is enriched in stromal components and blood vessels connected with the embryonic circulation by allantoic arteries and veins (Figure 1).

By 16 days of incubation, the CAM has grown so large that it completely covers most of the yolk sac and becomes adjacent to the shell membrane. The surface area of the CAM, which measures about 6 cm^2^ on day 6, increases to 65 cm^2^ by day 14 (76). The large surface extension and its position confer to the CAM a respiratory function through the pores in the eggshell (74).

As shown by Schlatter et al. (77), the CAM vasculature develops by both sprouting and intussusceptive angiogenesis in a three-phase process. In the first phase, multiple capillary sprouts invade the mesenchyme, fuse, and form the primary capillary plexus. During the intermediate phase, tissue pillars, expression of intussusceptive angiogenesis, replace capillary sprouts. In the third phase, the growing pillars increase in size to form intercapillary meshes [see (77, 78) for light microscopy and microvascular corrosion cast images of the three-phase process of the vascular development of the CAM].

In the early phase, the blood vessels are immature as they are not covered by smooth muscle cells and the basal lamina is incomplete. This initial structure allows the blood vessels to spread into the mesoderm, where they rapidly expand until day 8 to create a capillary plexus. The capillary plexus becomes close to the overlying chorionic epithelial cells, where it mediates gas exchange with the outer environment by receiving oxygen and eliminating carbon dioxide. Blood vessel proliferation continues until day 11. Then, it declines rapidly until day 18 when the vasculature attains its final arrangement up to hatching (79).

### The Chick Embryo Chorioallantoic Membrane for *in vivo* Studies on Angiogenesis

The CAM is a favored system for the *in vivo* study of physiological and pathological angiogenesis. Its extensive vascularization and easy accessibility make the CAM assay a simple experimental platform to investigate the efficacy and mechanisms of action of pro- and anti-angiogenic molecules. The assay is performed by grafting the materials to be tested onto developing CAM through a window cut in the eggshell. The embryogenesis starts as soon as the fertilized eggs are placed horizontally in an incubator at 37°C. The physiological environment for the CAM is guaranteed by working at controlled temperature and humidity. On day 3, after removing of approximately 5 ml of albumen, a window is opened in the shell to detach the CAM from the shell itself and to make the vascular surface accessible. This technique has the advantage of high viability in long-term incubation assays and allows the use of the embryos until just before hatching (at day 21), its disadvantages being represented by a limited area for manipulation and observation (22).

To avoid the disadvantage of the limited area of work, it is possible to transfer the embryo with its extraembryonic membranes into a Petri dish on day 3–4 of incubation. This experimental setting favors CAM development at the top of the Petri dish as a flat membrane on which multiple tests can be grafted (80). In addition, this *ex ovo* system is more suitable for live imaging than *in ovo* techniques and it allows the quantification of the response over a full area of the CAM by testing simultaneously a large number of samples. However, long-term viability is often shorter than *in ovo*, and more care is needed to avoid embryo dehydration. Usually, 50% of the *ex ovo* cultures is lost in the first 3 days after opening, due to the frequent rupture of the yolk membrane or to the sliding of the CAM at the bottom of the dish (80).

Several protocols have been developed for the release of molecules to be tested in the CAM assay. Macromolecules and low molecular weight compounds are placed onto the CAM using silostatic or silicon rings, methylcellulose disks, filters, plastic rings, or sponges. Sponges can be made in collagen or gelatin and are suitable also for testing the effects of cell xenografts (81). As compared to the direct delivery on the CAM of pure pro- or anti-angiogenic factors, the use of sponges loaded with a small number of cells allows the slow and continuous delivery of cell-secreted factors, thus mimicking a more “physiological” mode of interaction with the CAM vasculature.

Usually, an angiogenic response occurs 72–96 h after stimulation. The pro-angiogenic activity of a compound results in an increased blood vessel density around the implant, with newly formed blood vessels arranged in a radial pattern like the spokes of a wheel. On the contrary, when a compound with an anti-angiogenic activity is tested, the blood vessels become less numerous around the implant, and occasionally they disappear.

Different semi-quantitative and quantitative morphological and molecular methods have been developed to evaluate pro- or anti-angiogenic responses in the CAM assay at macroscopic and microscopic levels. Quantification of the CAM vasculature can be performed with the use of extensive vessel-counting methods based on visual examination and manual vessel counts or global measurements of the spatial pattern and distribution by algorithms. At the end of the assay, the membranes can be processed for in-depth analysis by immunohistochemistry preceded by paraffin embedding, or for ultrastructure analysis by electron microscopy.

Moreover, fresh CAM samples can be processed for molecular studies, including the determination of DNA amount, selected protein and collagen content (by Western blotting or spectrophotometric based-methods), and gene expression analysis by quantitative RT-PCR.

### The Chick Embryo Chorioallantoic Membrane for *in vivo* Studies on Inflammation

The immune system of the chick begins to develop during the embryonic life (82). Classically, innate responses are essential in the earliest phases of microbial invasion, until adaptive responses (B and T cell-mediated) become active to clear the infection. The chick immune system consists of B and T cells that control humoral and cell-mediated immunity, respectively. The B cells differentiate in the bursa of Fabricius, whereas T cells differentiate in the thymus (83, 84). The presence of T cells can be first detected at day 11 and of B cells at day 12 (85), and by day 18 chick embryos become immunocompetent (86, 87).

The first line of defense against bacterial pathogens in the chick embryo is represented by heterophils (88). These rounded cells release microbicidal agents, including ROS, proteolytic enzymes, and microbicidal peptides from their cytoplasmic granules. Heterophils present two types of granules. The primary granules are fusiform, display a central body that may be proteinaceous, and appear brick-red in color after Romanowsky stains. The secondary granules are rounded, less abundant, and smaller compared to the primary ones. Unlike mammalian neutrophils, chick heterophils are devoid of myeloperoxidase (88).

The chick embryo yolk sac produces the first generation of macrophages. Chick embryonic macrophages, identified at embryonic day 12–16 in the spleen and liver, recognize and phagocytize microbial antigens (89). In chickens, T-cell membrane protein 4 (TIM4) is a receptor expressed primarily by macrophages, binds to phosphatidylserine, and most likely participates in the recognition and clearance of apoptotic cells (89). Hu and colleagues applied anti-chicken TIM4 monoclonal antibodies in combination with colony stimulating factor 1 receptor reporter transgenes to dissect the function of TIM4 in the chick (90). They demonstrated that TIM4 was present on the large majority of macrophages during development *in ovo* and to be expressed also by other cells with phagocytic activity, such as dendritic cells, after hatching (90).

**Figure 2:**
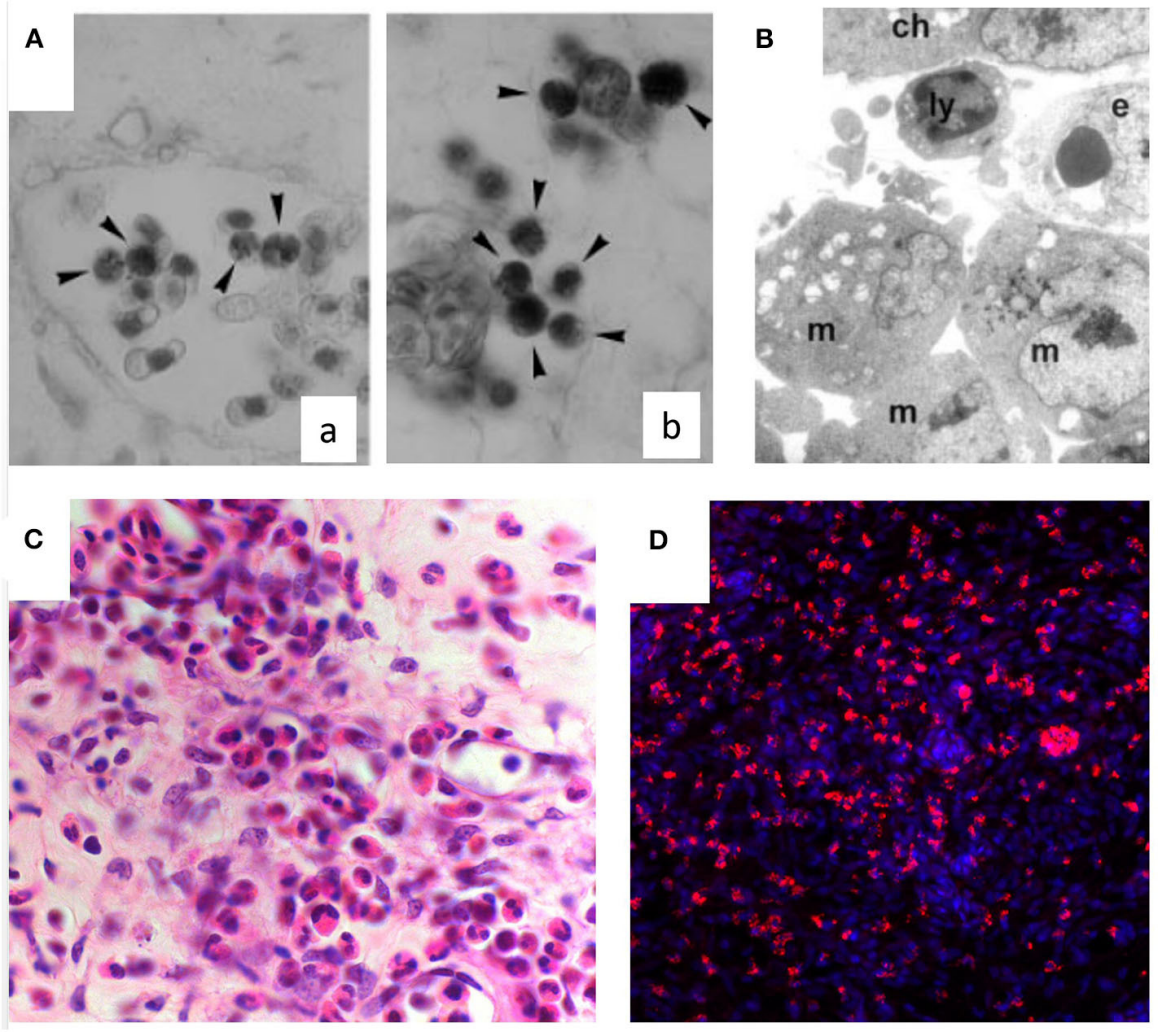
Inflammatory infiltrate in the chick embryo CAM. **(A)** Naphtol-AS-D-chloroacetate esterase-positive macrophages (arrowheads) in intravascular (a) and perivascular position (b) in the CAM mesoderm. **(B)** Macrophages (m) and a lymphocyte (ly) are recognizable at ultrastructural level around the endothelium (e) beneath the chorion (ch). **(C)** Histological sections of quartz filters implanted onto the CAM surface and stained with H&E. Note an increasing number of microvessels (arrows) and of the inflammatory infiltrate inside the marked area [reproduced from (91)]. **(D)** CD45^+^ macrophages (in red) infiltrating the CAM following treatment with PDR vitreous. Nuclear staining with DAPI (in blue).

An inflammatory response may be induced in the CAM assay through different stimuli. Inflammatory cells, first heterophils and then monocyte/macrophages, infiltrate the CAM mesenchyme (Figure 2). These cells can deliver several pro- and anti-inflammatory factors and cytokines, as well as important modifiers of the extracellular matrix [i.e., matrix metalloproteinases (MMPs]. Chick heterophils express MMP-9 (53), while monocyte/macrophages deliver MMP-13 to facilitate angiogenesis in a coordinated fashion (92).

A systematic study on the interplay between angiogenesis and inflammation, using different carrier materials placed on the CAM (e.g., glass fiber filters, viscose and gelatin sponges, agarose and polyacrylamide gels) have shown that the vascular reaction is also due, at least in part, to an inflammatory reaction induced by the presence of such foreign materials (93). The reactions induced by these materials were compared with those induced by natural egg materials (white eggshell membrane, coagulated albumen, and yolk). In all the cases, the CAM reacted with the proliferation of ectodermal cells, fibroblasts, and blood vessels, resulting in a highly capillarized granulation tissue. Accordingly, the CAM has been used as an *in vivo* model to study wound repair (94). This model consistently reproduces all the phases observed in adult wound healing, including re-epithelization, angiogenesis, inflammation, and fibronectin deposition, resulting in scar formation (94). Histological examination of the CAM during wound healing demonstrated hyperplasia of the chorionic epithelium in the area involved in the repair process, and inflammatory infiltrates consisting mainly of monocytes/macrophages positive to chloroacetate esterase (Figure 2A). The CAM has been used also as a model for the evaluation of inflammatory effects by tissue tolerable plasma for the determination of the optimum parameters for treatment of chronic wounds. The response patterns, represented by granuloma development (with associated angiogenesis), hemorrhages, coagulation, and contracture, were alleviated when hydrocortisone was added immediately after plasma treatment (95). Hyaluronic acid/bone substitute complexes implanted on the CAM induce instead osteoblastic differentiation and angiogenesis, but not inflammation, while a massive inflammatory infiltrate was detected around the implant of hyaluronic acid and saline samples (96).

The presence of a mononuclear cell infiltrate has been observed also in osteopontin (OPN)-treated CAMs and responsible, at least in part, for the neovascular response triggered by this cytokine (60). Mononuclear cells were frequently found to encircle microvessels located at the boundary between the OPN-loaded sponges and the surrounding CAM mesenchyme, and the presence of mononuclear cells and lymphocytes has also been demonstrated at the ultrastructural level (60). Similarly, Andrés and colleagues demonstrated that FGF2-loaded alginate beads trigger a robust angiogenic response when implanted on the CAM surface (97). In parallel, the presence of an inflammatory cell infiltrate in the stroma among the newly formed blood vessels was revealed by May Grünwald-Giemsa staining of the treated membranes. Furthermore, to prove the non-redundant role of the inflammatory cells/mediators in FGF2-dependent neovascularization, the experiments were repeated in the presence of hydrocortisone and ketoprofen drugs. The results showed that both drugs were able to inhibit the angiogenic response triggered by FGF2 (97). In this frame, Sung et al. examined the *in vivo* effects of the sequential delivery of dexamethasone followed by VEGF on the immune response and vascular network formation in the CAM assay. Cross-section images of control CAMs showed very few inflammatory cells, mostly macrophages and heterophils. In contrast, an abundant presence of inflammatory cells, fibroblast encapsulation, and swelling (edema) were found in the tissue surrounding the VEGF implant that were inhibited by dexamethasone (98).

Together, these data indicate that the chick embryo CAM represents a platform suitable for the study of the cross talk between angiogenesis and inflammation.

## The Chick Embryo Chorioallantoic Membrane for Diabetic Retinopathy Studies

The use of the chick embryo CAM for the study of retinal vascular pathologies dates back to the early' 80s. Glaser and colleagues utilized the CAM to investigate the vasoproliferative activities of several mammalian tissue extracts (i.e., liver, cardiac skeletal muscle, and retina). They observed a potent vasoproliferative response when pellets containing retinal extracts were applied on the top of the CAM, while other adult tissues resulted ineffective (99). With a similar approach, Okamoto and colleagues demonstrated that extracts derived from rabbit retina, iris-ciliary body, and optic nerve exerted an angiogenic activity on CAM, with retinal extracts inducing the strongest effect (100). On these bases, the CAM assay was applied for testing angiogenic factors extracted from both cat and bovine retinas (101), and Prost compared the angiogenic activity of the detached retina with that of the normal attached retina, demonstrating that the detached retina exhibits a stronger angiogenic activity (102). The first experimental evidence that the CAM assay could provide useful information for the study of DR was obtained by Hill and colleagues. In this study, the vitreous humor from PDR patients promoted the proliferation of CAM blood vessels, while vitreous from non-diabetic patients was ineffective (103). Thereafter, Taylor et al. isolated an endothelial cell-stimulating angiogenic factor from the human vitreous and demonstrated its pro-angiogenic activity in the CAM assay (104). In addition to neovascular studies, the CAM has been used as a substrate for maintaining mammalian retinal explants in culture (105) and as a model for testing novel surgical procedures for cutting and coagulating the retinal vasculature (106). More recently, the CAM has represented a platform to evaluate the pro-angiogenic/pro-inflammatory activity of the humor vitreous obtained from PDR patients.

### The Chick Embryo Chorioallantoic Membrane and PDR Vitreous Humor

Vitreous humor obtained via *pars plana* vitrectomy from PDR patients has been shown to exert significant biological responses when delivered *in vitro* and *in vivo* to different cell types in various pre-clinical experimental models [reviewed in (107)]. Thus, the study of the biological activity of PDR vitreous may provide further insights into the relationship between inflammation and angiogenesis. It has been demonstrated that PDR vitreous contains high levels of both pro-inflammatory and pro-angiogenic factors (17, 108). As a consequence, the biological activity exerted by PDR vitreous in *in vitro* and *in vivo* models depends on the balance between all the mediators that have accumulated in the ocular fluid during the progression of the disease and that are present at time of harvesting. Moreover, PDR vitreous can be employed in several experimental models in order to screen and characterize drugs with potential pharmacological applications.

In this frame, we have shown that PDR vitreous induces a pro-angiogenic response in endothelial cells whereas vitreous fluid obtained from macular hole patients was ineffective (109–114). Indeed, PDR vitreous fluid activates *in vitro* all the steps of the angiogenic process, including endothelial cell proliferation, motility, sprouting, and tube formation. At the same time, PDR vitreous induces a pro-inflammatory activation of endothelial cells characterized by the nuclear translocation of the pro-inflammatory transcription factors NF-κB and CREB, ROS production, disruption of endothelial intercellular junctions, upregulation of the cell adhesion receptors vascular cell adhesion protein 1 and ICAM-1 and consequent increase of leukocyte adhesion.

**Figure 3:**
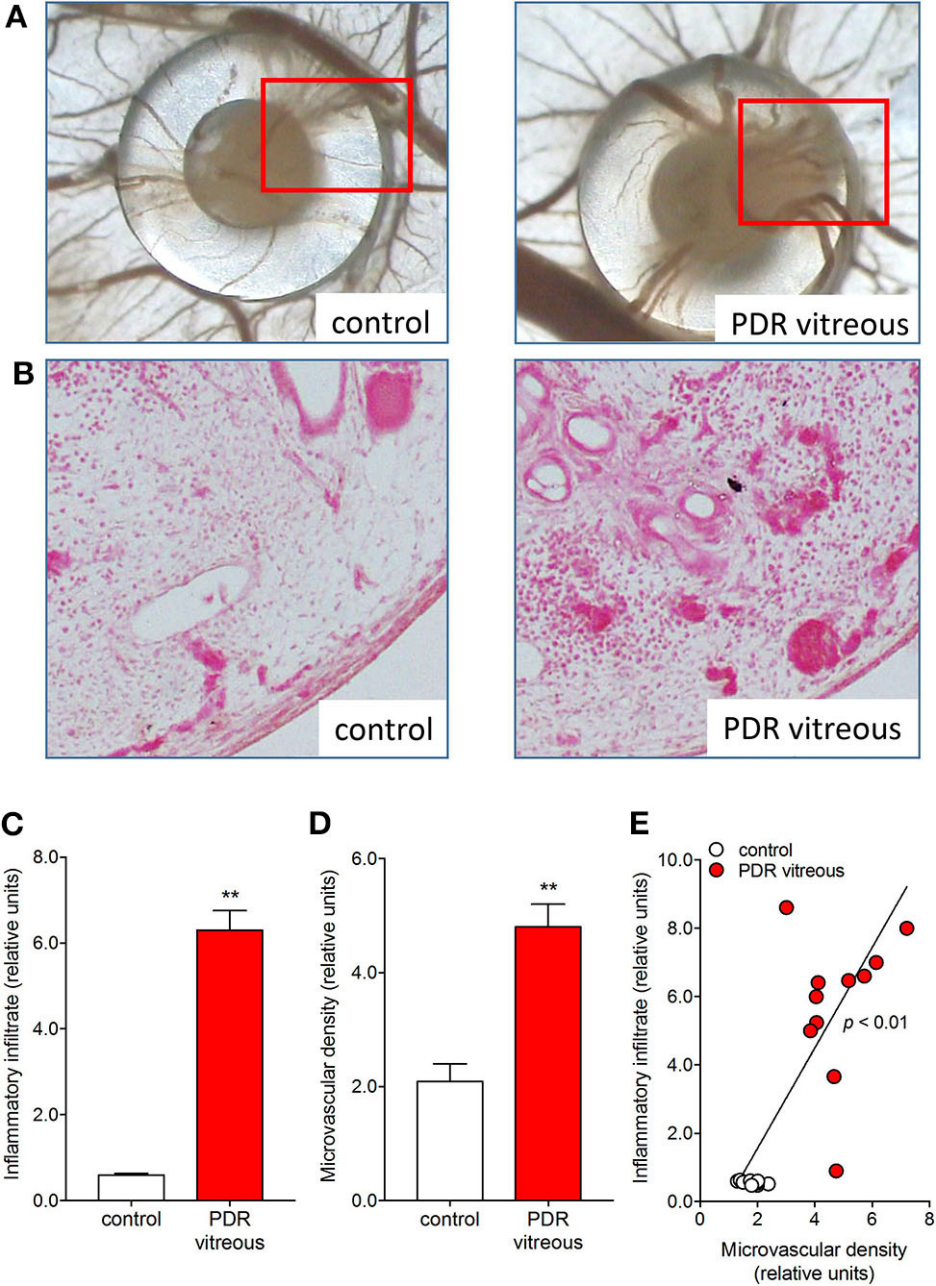
PDR vitreous induces an angiogenic/inflammatory response in the chick embryo CAM. **(A)** Macroscopic pictures of the CAM at day 12 of incubation, showing a silicon ring containing vehicle (control) and a PDR vitreous sample. Note a strong angiogenic response in the experimental sample as compared to the control one. **(B)** Histological sections of the marked areas evidenced in **(A)**. Note a strong angiogenic response and a dense inflammatory reaction in the experimental sample as compared to the control one. **(C, D)** Morphometric quantification of the inflammatory infiltrate area (C) and of the microvascular density area **(D)**. **(E)** Correlation between microvascular density and inflammatory infiltrate induced by PDR vitreous in the CAM assay. **p < 0.01 *v*s control, Student's *t* test.

In keeping with these *in vitro* data, the chick embryo CAM assay provided useful *in vivo* information about the pro-angiogenic/pro-inflammatory activity of PDR vitreous. Alginate beads loaded with 2.0 μl/pellet of a pool of vitreous samples obtained from PDR patients were engrafted onto the surface of the chick embryo CAM at 11 days of development. After 72 h, several neovessels moving toward the graft were detected. Moreover, the beads containing PDR vitreous attracted a significant population of mononuclear cells, which was absent in controls (115). Significantly, the number of neovessels was correlated with the extent of the inflammatory infiltrate (Figure 3).

**Figure 4:**
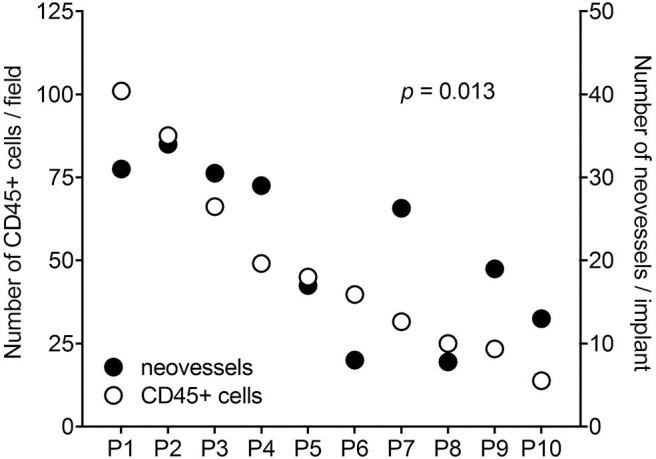
Correlation between the angiogenic and inflammatory responses triggered by individual PDR vitreous samples in the chick embryo CAM. Vitreous samples from 10 PDR patients were individually tested in the CAM assay. A significant relationship was observed between the number of neovessels and of CD45^+^ infiltrating cells induced by each vitreous sample.

It is worth noticing that a high variability in the angiogenic and inflammatory responses was observed when vitreous samples obtained from 10 patients with PDR were individually applied to the top of the CAM (115). This may be the consequence of the individual medical case history and clinical features of PDR patients, resulting in a significant qualitative and quantitative heterogeneity in the composition of pro-inflammatory/pro-angiogenic mediators present in the vitreous fluid at the last stages of the disease. Nevertheless, also in this case a significant correlation was observed between the number of infiltrating CD45+ cells and the number of new blood vessels elicited by PDR vitreous samples in the CAM assay (Figure 4). Since the more angiogenic samples were able to trigger a more significant inflammatory response, these data support the notion that angiogenesis and inflammation are closely related processes during PDR. Accordingly, treatment with hydrocortisone was able to reduce drastically the angiogenic response and the recruitment of inflammatory cells induced by PDR vitreous in the CAM assay. Thus, inflammation appears to play a significant role in the angiogenic activity exerted by PDR vitreous.

*N*-formyl peptide receptors (FPRs) belong to a class of pattern recognition receptors that are involved in controlling inflammation, angiogenesis, tissue repair, and innate immune responses (116). The tetrapeptide Ac-L-Arg-Aib-L-Arg-L-Cα(Me)Phe-NH2 (UPARANT) blocks urokinase-type plasminogen activator receptor (uPAR)-dependent cell signaling by interfering with the complex cross-talk among FPRs, uPAR, and integrins. Accordingly, UPARANT competes with *N*-formyl peptides for the binding to FPRs and inhibits VEGF-driven angiogenesis by preventing FPR activation (117). Recent studies have shown that UPARANT exerts an anti-angiogenic and anti-inflammatory activity when tested in animal models of oxygen-induced retinopathy by inhibiting ocular neovascularization and by lowering the levels of inflammatory molecules (115). Accordingly, UPARANT successfully inhibited the formation of novel blood vessels promoted by 16 out of 20 individual samples of PDR vitreous in the CAM assay. Again, its anti-angiogenic effect was linearly correlated with a reduced inflammatory infiltrate, suggesting that FPR activation may play a non-redundant role in promoting neovascularization during PDR (115).

Three FPRs have been identified in humans (FPR1–FPR3), characterized by different ligand properties, biological function and cellular distribution (118). Among them, FPR3 appears to mediate pro-angiogenic responses in human endothelial cells (119). It must be pointed out that the murine genome contains eight FPR-related sequences (120) whereas the presence of FPR gene ortholog(s) in birds is more uncertain. Indeed, a cell surface protein immunoreactive with a specific anti-human FPR1 antibody is detectable in chick embryo neurons and glial cells and BLAST search has identified numerous putative *N*-formyl peptide receptors in the avian genome. However, experimental evidences suggest that these receptors might be identified with members of the chemokine receptor CXCR4 subfamily able to act as *N*-formyl peptide binders (121). Thus, caution should be taken before extrapolating the results obtained in animal models, including the CAM, about the possible impact of FPRs on the angiogenic process in humans.

**Figure 5:**
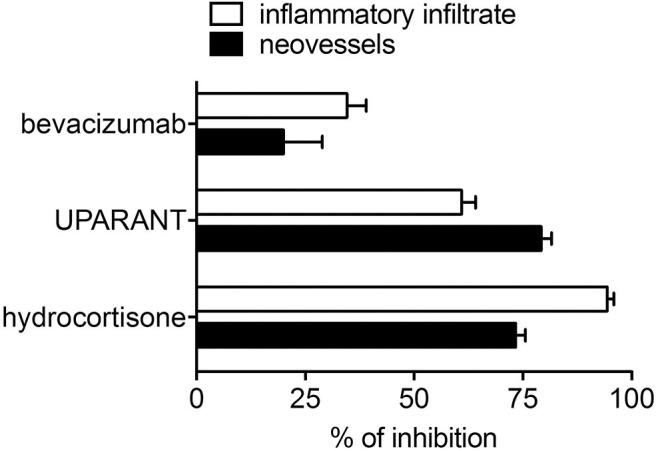
Inhibition of the angiogenic/inflammatory response induced by PDR vitreous in the chick embryo CAM. Chick embryo CAMs were treated with PDR vitreous in the absence or in the presence of different inhibitors. Note the more potent inhibitory effect exerted by the anti-inflammatory agents hydrocortisone and UPARANT when compared to the anti-VEGF drug bevacizumab.

Notably, unlike the anti-inflammatory agents hydrocortisone and UPARANT, the anti-VEGF drug bevacizumab induces only a moderate inhibition of neovascularization and inflammatory cell recruitment promoted in the CAM assay by PDR vitreous-loaded beads [see Figure 5 and (115)]. The limited efficacy of bevacizumab may depend on the presence of several other pro-inflammatory and/or pro-angiogenic cytokines and growth factors in addition to VEGF, which contribute to the biological activity of PDR vitreous. In keeping with this hypothesis, the biotechnological heparin-like molecule K5-N,OS(H), endowed with the capacity to bind several heparin-binding inflammatory and/or angiogenic mediators present in PDR vitreous, have shown a potency much stronger than bevacizumab in inhibiting the angiogenic response elicited by PDR vitreous (109).

Taken together, these data suggest that the pro-angiogenic and pro-inflammatory activity of PDR vitreous may depend on the synergistic action of multiple molecules, supporting the belief that inflammation and angiogenesis may be strictly correlated, with inflammation being a relevant factor in the formation of novel retinal blood vessels during PDR.

## Concluding Remarks

The chick embryo CAM assay presents numerous advantages, such as its low cost, reproducibility and reliability, and simplicity in execution. Furthermore, in most countries the use of chick embryo until day 17 of development is not subjected to regulatory rules in order to obtain ethics committee approval for animal experimentation.

As described in this review, recent experimental evidence has shown that the vitreous obtained from PDR patients elicits angiogenic and inflammatory responses when delivered on the top of the CAM. Notably, despite the fact the PDR vitreous samples are collected after pars plana vitrectomy at the end stage of the disease, when no other therapeutic innervations are available, individual samples are characterized by a highly variable biological effect when tested in the CAM assay. Such variability has been observed also in *in vitro* experiments when the same samples were tested on cultured endothelial cells. These data indicate that such variability does not represent a drawback of the CAM assay but it rather reflects an individual heterogeneity among PDR patients, possibly related to differences in their medical case history and clinical features that result in a different angiogenic/inflammatory profile. Nevertheless, despite this heterogeneity, a significant direct correlation has been observed between the extent of neovascular and inflammatory responses elicited by PDR vitreous samples in the CAM assay, strengthening the concept that a tight correlation indeed exists between angiogenesis and inflammation in PDR. This concept is supported by the observations that different anti-inflammatory agents hamper the angiogenic activity exerted by PDR vitreous, as well as by recombinant growth factors/cytokines.

The clinical observation that anti-VEGF therapies may show only a limited effect in PDR patients calls for new pharmacologic interventions. New insights into the impact of inflammation in the pathogenesis of PDR may allow the discovery of novel therapeutic targets. The association of anti-angiogenic and anti-inflammatory drugs may therefore be beneficial for treating PDR. In this frame, the CAM assay may represent a suitable platform for a rapid *in vivo* screening of novel drug candidates.

A critical limitation in the use of the CAM for *in vivo* studies may be the lack of avian-specific reagents, as well as the presence of species-specific differences and the insufficient genomic information. However, the usage of retroviral, adenoviral, and lentiviral vectors has been applied to the infection of the CAM, making them express a long-lasting viral transgene. This technique has been employed for studying dominant-negative gene products, as well as for evaluating the effects of intracellular or membrane-bound proteins. In addition, the achievement of the chick embryo genome sequencing (122) should support the synthesis of a broad panel of antibodies with high specificity for chick cells and stroma components.

**Figure 6:**
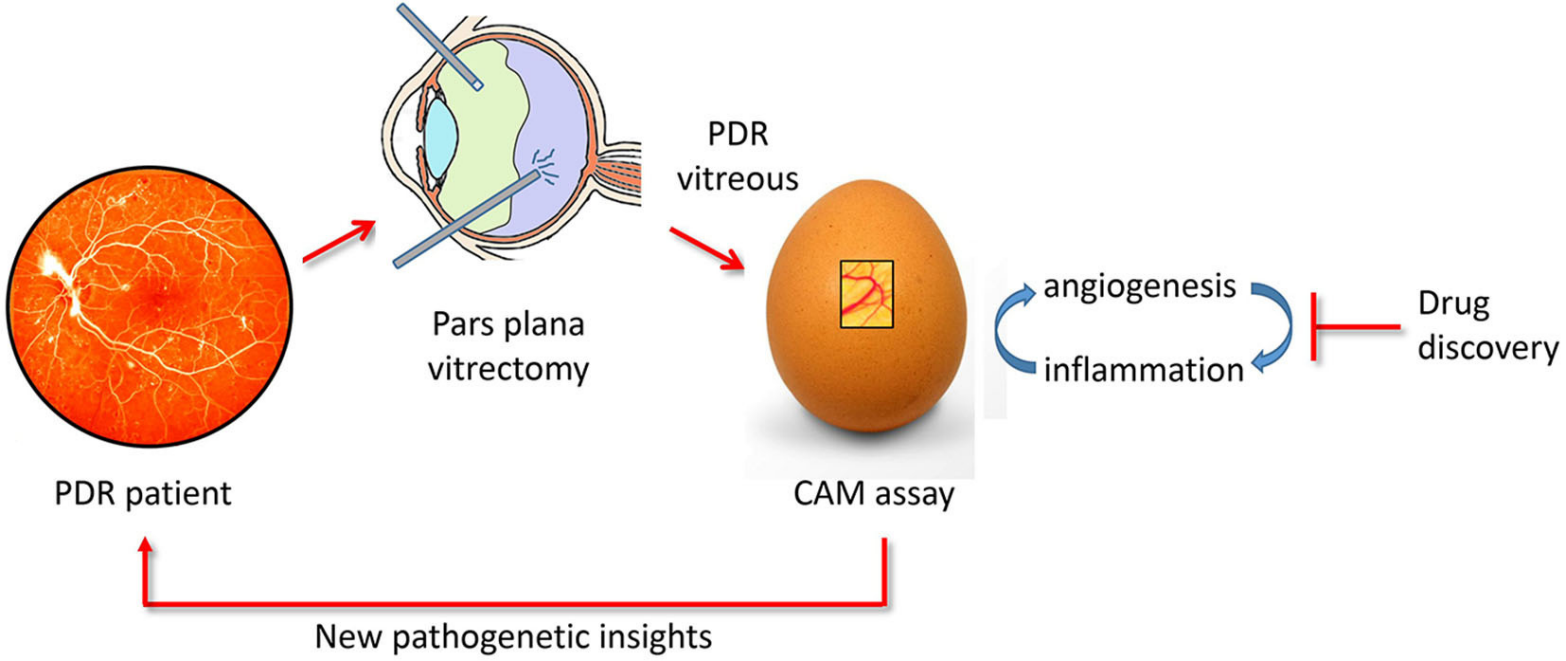
The chick embryo CAM/PDR vitreous platform. PDR vitreous obtained by *pars plana* vitrectomy provides a useful tool for drug discovery when tested in the CAM assay. In addition, the study of the cross talk between the angiogenic and inflammatory responses elicited by PDR vitreous in the CAM assay may shed a new light on the pathogenesis of the disease.

In conclusion, the CAM assay may represent a cost-effective and rapid tool for the study of the relationship between neovascular and inflammatory responses elicited in PDR and for the screening of novel therapeutic agents (Figure 6).

## Author Contributions

MP revised and redacted the final version. All authors contributed to the writing of the manuscript.

## Conflict of Interest

The authors declare that the research was conducted in the absence of any commercial or financial relationships that could be construed as a potential conflict of interest.
